# Overexpression of *pdeR* promotes biofilm formation of *Paracoccus denitrificans* by promoting ATP production and iron acquisition

**DOI:** 10.3389/fmicb.2022.966976

**Published:** 2022-08-10

**Authors:** Na Wang, Jie Gao, Shujie Xiao, Guoqiang Zhuang

**Affiliations:** ^1^CAS Key Laboratory of Environmental Biotechnology, Research Center for Eco-Environmental Sciences, Chinese Academy of Sciences, Beijing, China; ^2^Jinan Microecological Biomedicine Shandong Laboratory, Jinan, China; ^3^College of Resources and Environment, University of Chinese Academy of Sciences, Beijing, China

**Keywords:** biofilm, quorum sensing, PdeR, RNA-seq, *Paracoccus denitrificans*

## Abstract

Bacterial biofilms are ubiquitous in natural environments and play an essential role in bacteria’s environmental adaptability. Quorum sensing (QS), as the main signaling mechanism bacteria used for cell-to-cell communication, plays a key role in bacterial biofilm formation. However, little is known about the role of QS circuit in the N-transformation type strain, *Paracoccus denitrificans*, especially for the regulatory protein PdeR. In this study, we found the overexpression of *pdeR* promoted bacterial aggregation and biofilm formation. Through RNA-seq analysis, we demonstrated that PdeR is a global regulator which could regulate 656 genes expression, involved in multiple metabolic pathways. Combined with transcriptome as well as biochemical experiments, we found the overexpressed *pdeR* mainly promoted the intracellular degradation of amino acids and fatty acids, as well as siderophore biosynthesis and transportation, thus providing cells enough energy and iron for biofilm development. These results revealed the underlying mechanism for PdeR in biofilm formation of *P. denitrificans*, adding to our understanding of QS regulation in biofilm development.

## Introduction

Biofilms are one of the most prevalent and important forms of life for bacteria, in which cells are encased in the extracellular matrix that can serve as a barrier to multiple adverse environmental factors (Steenackers et al., 2016; Jo et al., 2022). Previous studies showed that the environmental stresses that microbes would face can be highly variable and complex, including mechanical damage, antibiotics pressures, oxidative stresses, etc. (Starosta et al., 2014). Thus, over 80% of bacteria would form a biofilm to resist environmental stresses and to make sure the whole community survives better (Mohammad Reza, 2018). For example, under extreme conditions such as the deep sea, microorganisms would form microbial mats and produce more extracellular polymeric substances (EPS) to resist mechanical stress (Bolhuis et al., 2014). Moreover, EPS could work as a protective barrier, which contributes to the lower sensitivity and higher resistance of biofilms to antibiotics (Hoiby et al., 2010; Yan and Bassler, 2019), and biofilm also promotes the efficiency of resistance genes horizontal transfer (Orazi and O’Toole, 2019). Typically, biofilm formation and maturation are regulated by various factors, including cell-to-cell communication and environmental factors.

Quorum sensing (QS) is a widely used bacterial communication mechanism, by which bacteria could secret and sense signaling molecules called autoinducers to coordinate gene expression according to the population density (Papenfort and Bassler, 2016; Mukherjee and Bassler, 2019). Bacteria use QS to precisely coordinate various of group behaviors, including biofilm formation, carbon metabolism, EPS production, virulence factors production, luminescence, etc. (Davies et al., 1998; Hense and Schuster, 2015). Previous studies have shown that the mutation of QS system severely damaged the ability of biofilm formation, such as in *Pseudomonas aeruginosa*, *Burkholderia cepacia*, *Streptococcus mutans*, etc. (Lewenza et al., 1999; Parsek and Greenberg, 2005). QS coordinates the initiation and maturation of biofilm by regulating the expression of a series of functional genes, including EPS production related genes, cell motility genes, and so on. Gilbert et al. demonstrated that the QS regulation protein LasR in *P. aeruginosa* could directly regulate the expressional level of the extracellular polysaccharide (Psl) biosynthetic genes (Gilbert et al., 2009). Moreover, when the second QS circuit *rhl* was mutant, the production of another kind of polysaccharide Pel significantly decreased in *P. aeruginosa* (Sakuragi and Kolter, 2007). In addition, QS could coordinate the expression of cell motility genes, such as IV pilus gene clusters which are associated with cell attachment during the early stage of biofilm formation, as well as the genes for flagella synthesis which are essential for biofilm matures as a mushroom-like structure (Yu and Ma, 2017; Yan and Bassler, 2019). However, research on the mechanism of QS regulating biofilm formation is mostly concentrated in pathogenic microorganisms, such as *P. aeruginosa*, *Staphylococcus aureus*, etc., while studies on environmental bacteria are relatively rare.

*Paracoccus denitrificans* is widely distributed in soil and water, possessing the ability of heterotrophic nitrification-aerobic denitrification (HNAD), and thus is taken as the model strain for nitrogen transformation research (Ji et al., 2015). We have demonstrated that *P. denitrificans* harbored a LuxI/R-type QS circuit, PdeI/R (Zhang et al., 2018). The AHL synthetase PdeI could catalyze the biosynthesis of *N*-hexadecanoyl-L-Homoserine lactone (C16-HSL), and PdeR protein is the corresponding regulatory protein which could bind with C16-HSL and regulate genes expression. It has been shown that *P. denitrificans* forms a peculiarly thin biofilm at the gas–liquid interface, which consisted of almost a monolayer of cells (Yoshida et al., 2017). Biofilms for *Paracoccus* species have many important applications, especially in wastewater nitrogen removal bioreactors, while the detailed mechanism of biofilm formation in this genus is largely unknown (Morinaga et al., 2020). Previous studies showed that in the *pdeI* mutant strain, cell aggregation was more obvious and bacteria formed thicker biofilm, while exogenous C16-HSL addition inhibited cell aggregation (Morinaga et al., 2018). Nevertheless, the underlying mechanism of QS regulating biofilm formation in *P. denitrificans* still remains unclear, especially for the role of PdeR protein.

In this study, we constructed the *pdeR* overexpression strain to explore the role of PdeR on biofilm formation of *P. denitrificans* PD1222 at the initiation stage. The results of physiological tests indicated overexpressed *pdeR* promoted cell aggregation and EPS production, and thus the *pdeR* overexpression strain formed thicker biofilm. Furthermore, through transcriptomic analysis as well as biochemical experiments, we demonstrated that PdeR promotes adenosine triphosphate (ATP) production and iron absorption during the initial stage of biofilm formation to provide sufficient energy and iron for biofilm development. The results of this study deepened our understanding of the QS regulation mechanism for biofilm formation of *P. denitrificans*, providing some useful references for the optimization of *P. denitrificans* in applications.

## Materials and methods

### Bacterial strains, plasmids, and growth conditions

Bacterial strains and plasmids used in this study are listed in Table 1. *P. denitrificans* and its derived strains were cultivated at 30°C, 180 rpm. *Escherichia coli* and its derived strains were cultivated at 37°C, 180 rpm. All strains were grown in Luria-Bertani (LB) medium, if necessary, supplied with rifampicin 50 μg/ml, kanamycin 100 μg/ml, ampicillin 100 μg/ml, or chloramphenicol 34 μg/ml to maintain plasmids and select for recombinants.

**Table 1:** Strains and plasmids used in this study.

Strain or plasmid	Relevant characteristic (s)^a^	Source or references
Strains		
*Paracoccus denitrificans*		
PD1222	Wild type, G^−^, Rif^r^	Zhang et al., 2018
PD-pdeR	PdeR overexpression strain, Rif^r^, Kan^r^	This study
PD-pBBR	PD1222 containing empty pBBR1MCS, Rif^r^, Kan^r^	This study
*Escherichia coli*		
DH5α	Host strain for pBBR-pdeR, pBBR1MCS-2; used as donor in triparental conjugation	Takara
Plasmids		
pBBR1MCS-2	Broad-host-range cloning vector, Kan^r^	Kovach et al., 1995
pRK600	Helper plasmid, mob^+^ tra^+^, Cm^r^	Lab stock
pBBR-*pdeR*	Constructed overexpression vector, Kan^r^	This study
pKK223-3	Used to amplify the Tac promoter, Amp^r^	Lab stock

a G^−^, Gram negative; Rif^r^, rifampin resistant; Kan^r^, kanamycin resistant; Cm^r^, chloramphenicol resistant; mob^+^, plasmid mobility; tra^+^, plasmid transferability; Amp^r^, ampicillin resistant.

### Construction of a *pdeR* overexpression strain

The fragment of *pdeR* gene (Pden_0786) was amplified *via* PCR from the genome of *P. denitrificans* PD1222 with the addition of a 6 × His tag and then fused with a *tac* promoter (5′-GTGTGGAATTGTGTTGACAATTAATCATCGGCTCGTATAATGTGTGGAATTGTG-3′). Primers used in this study are listed in Table 2. The obtained *tac*-*pdeR*-His fragment and the broad-host-range plasmid pBBR1MCS-2 were all double digested by AgeI (NEB, United States) and XbaI (NEB) in 37°C, 2 h. Ligations were performed overnight at 16°C, using T4 DNA ligase (NEB). The constructed plasmid pBBR-*pdeR* was then introduced into PD1222 by triparental conjugation. Briefly, PD1222 was used as the recipient, *E. coli* DH5α harboring pBBR-*pdeR* was used as a donor, *E. coli* DH5α harboring pRK600 was used as a helper, and strains were mixed with the ratio of 3:1:1. After being cultivated in a fresh LB agar plate for 24 h, the mixture was spread on the plate supplemented with rifampin (100 μg/ml) and kanamycin (50 μg/ml) to select *pdeR* overexpression strain PD-pdeR. And the control strain PD-pBBR that harbors the empty plasmid pBBR1MCS-2 was constructed in the same way.

**Table 2:** Primers used for plasmid construction.

Primer name	Primer sequence (5′–3′)
pKK-1	GCTCTAGACAAGGCGCACTCCCGTTCTGGATAATG
pKK-RH-2	CTTGGCGAGTGCTGCATTGATTTCCGCGCGAGACGACATTTCTGTTTCCTGTGTGAAATTGT
pKK-RH-3	GTGTGGAATTGTGAGCGGATAACAATTTCACACAGGAAACAGAAATGTCGTCTCGCGCGGAAATCAAT
R-His	GCACCGGTTTAATGATGATGATGATGATGCAGCAAGCGGTAATCCTTGGC
pden-NC-F	TCGGAATTACTGGGCGTAAAG
pden-NC-R	TCGAACTCCAGACCGATAGT
P0786-11	ATCACGGCCTGCACTATG
P0786-12	CGATCTCGTCATCCGTGAAT

### Biofilm and EPS characterization

Crystal violet staining was performed as previously described to characterize the difference in biofilm formation between PD-pdeR and PD-pBBR with some modifications (Wang et al., 2019). Cultures of PD-pdeR and PD-pBBR were incubated in polystyrene 12-well microtiter plates at 30°C, 180 rpm. Cells were collected after 12, 24, and 36 h incubation, respectively. The cell suspensions were decanted and the remained cells were softly washed by PBS buffer three times and then fixed at 60°C for 10 min. The attached cells were quantified by staining with 0.1% (*w*/*v*) crystal violet solution for 15 min, and then washed three times with PBS and air dried. The bound crystal violet was extracted using 95% ethanol. A spectrophotometer (PerkinElmer, EnSpire2300) was used to detect the absorbance at 595 nm. Three parallel experiments were carried out.

The cationic exchange resin method was used to extract EPS as previously described (Wang et al., 2022). Briefly, cells after 36 h incubation were collected by centrifuging at 8,000 *g* for 10 min, and resuspended again in sterile water. Cationic exchange resin (0.25 g/ml) was added to the suspension and transferred to a conical flask to extract EPS. The conical flasks were put in a shaking incubator at 200 rpm overnight at room temperature. Then the suspension was centrifuged at 10,000 *g* for 10 min at 4°C and the supernatant was filtered through 0.45 μm filter. The content of exopolysaccharides was determined by the phenol sulfuric acid method (Dubois et al., 1956), and glucose solutions were used for the standard curve. The content of extracellular protein was measured using the Pierce^™^ Coomassie Assay Kit (Thermo Fisher Scientific, United states), using bovine serum protein as the calibration curve standard.

### Scanning electron microscope observation

PD-pBBR and PD-PdeR have incubated in polystyrene 48-well microtiter plates in which cover glasses were pre-placed in each well. After 36 h, cell suspensions were carefully removed and the remaining cells adhered to the slides were fixed with 2.5% glutaraldehyde overnight at 4°C. The fixed slides were washed three times in PBS buffer and dehydrated using 50, 70, 80, 90, and 100% ethanol solutions (15 min at each gradient). After lyophilized for 2 h using a freeze–drying device, the slides were sprayed with gold and observed by a scanning electron microscope.

### Real-time quantitative PCR

Cells of PD1222, PD-pBBR and PD-pdeR were harvested when OD_600_ was about 1.5. Total RNAs were isolated and purified with an RNeasy mini system (Qiagen, Germany) and then reverse transcribed by PrimeScript^™^RT Master Mix (Takara, Japan) according to manufacturer’s instructions. Quantitative PCR reactions were performed using the SYBR^®^ Premix Ex Taq Kit (Takara, Japan) and a 7,500 real-time PCR system (Bio-Rad, United States). The primers used in Real-time Quantitative PCR (RT-qPCR) analyses were designed by Primer premier 5 and are listed in Table 2. The relative expression of *pdeR* was calculated by the 2^–ΔΔCT^ method (Livak and Schmittgen, 2001) with 16 s rRNA of *P. denitrificans* used as an internal control. All reactions were carried out in triplicate.

### Transcriptome analysis

Triplicate cultures of PD-pBBR vector and PD-pdeR were grown in LB medium at 30°C, 180 rpm. When the OD_600_ reached ~ 1.5, cells were harvested, and total RNAs were extracted. The total RNA extraction of PD-pBBR and PD-pdeR was performed by using the RNeasy Mini kit (Qiagen, Germany), treated with DNase I (Qiagen, Germany). The quality of isolated RNAs was analyzed by electrophoresis and quantified by a NanoDrop 2000 spectrophotometer (NanoDrop Technologies). And then the RNAs were subjected to Solexa/Illumina sequencing at Beijing Auwigene Tech.

The clean data were obtained by removing reads that contained possible sequencing errors and were mapped to the downloaded reference genome sequences of *P. denitrificans* PD1222 using Bowtie2. The relative transcript abundance was measured by FPKM (Fragments per kilobase of exon model per million mapped reads). The criteria for differentially expressed genes screening was p. adjust value of less than 0.05. Furthermore, the functional enrichment analyses including gene ontology (GO) and Kyoto Encyclopaedia of Genes and Genomes (KEGG), were performed to dig out the biological function of differentially expressed genes (DEGs).

### Acetyl-CoA and ATP quantitation

Cells of PD-pBBR and PD-pdeR were harvested when OD_600_ was about 1.5 by centrifugation at 4°C, 10,000 *g* for 5 min, and then resuspended in extraction buffer (methanol: acetonitrile: water = 45:45:10, *v*:*v*:*v*). Cells were then broken by ultrasonication (power 40%, 2 s-working, 1 s-pause, total time 10 min). The lysates were centrifuged at 14,000 *g*, 4°C for 10 min to remove the cell debris.

For acetyl-CoA extraction, add ice-cold perchloric acid (final concentration, 1 M) into the supernatant, and incubated on ice for 5 min. Then centrifuged at 4°C, 13,000 *g* for 2 min, transfer the supernatant to a new EP tube, adding ice-cold KOH solution to make sure its final concentration is 34%, then vortexed to mix the contents. Then adjust the pH value to 6.5–8 using 0.1 M KOH, 4°C, 13,000 *g*, centrifuged for 15 min, and transfer the supernatant to a new tube. The quantitation of acetyl-CoA was performed using PicoProbe Acetyl CoA Assay Kit (Abcam, United Kingdom), according to the manufacturer’s instructions. And the fluorescent intensity was measured using a SpectraMax i3× microplate reader (Ex/Em = 535/587 nm).

For ATP detection, adding 500 μl chloroform to per mL cell lysates, mixed well then centrifuged at 4°C, 10,000 *g* for 3 min, and collected the supernatant. The quantitation of ATP was performed using ATP Detection Kit (Solarbio, China). The operation was done according to the kit instructions. And samples were detected by absorbance at 340 nm.

### Siderophore detection

Arnow test was used to quantify the catechol siderophore followed the previous method with minor changes (Arnow, 1937). Briefly, bacterial cultures were collected after 12, 24, 36 and 48 h incubation by centrifugation at 10,000 *g* for 5 min. 1 ml of bacterial culture supernatant was mixed with 1 ml of 0.5 M HCl solution, 1 ml of nitrite-molybdate reagent, and 1 ml of 1 M NaOH solution in order. Mix thoroughly and add water to make 5 ml, incubated at room temperature for 10 min. The mixture solution was measured using a spectrophotometer (PerkinElmer, EnSpire2300) at 510 nm. All detective assays were carried out in triplicate.

### 2,3-Dihydroxybenzoic acid addition experiment

The 2,3-dihydroxybenzoic acid (DHBA) was purchased from Sigma-Aldrich. DHBA was first formulated into a mother liquor of 1 mg/ml and 10 mg/ml, then filter sterilized. The DHBA solutions were added into LB medium to make sure the final-concentrations were 1 μg/ml and 10 μg/ml, respectively. Same volume of sterile water was added as control. PD-pBBR and PD-pdeR were seeded in the medium with different concentrations of DHBA, and incubated for 36 h and then the biofilm formation was detected.

### Statistical analysis

All tests in this study were carried out in triplicate, and the results are presented as means ± SD (standard deviation of means). The significance among groups was analyzed by one-way ANOVA test (*p* < 0.05 were considered as significant) using the SPSS Statistics 24.0 software.

## Results

### Bacterial growth and morphological characteristics

**Figure 1:**
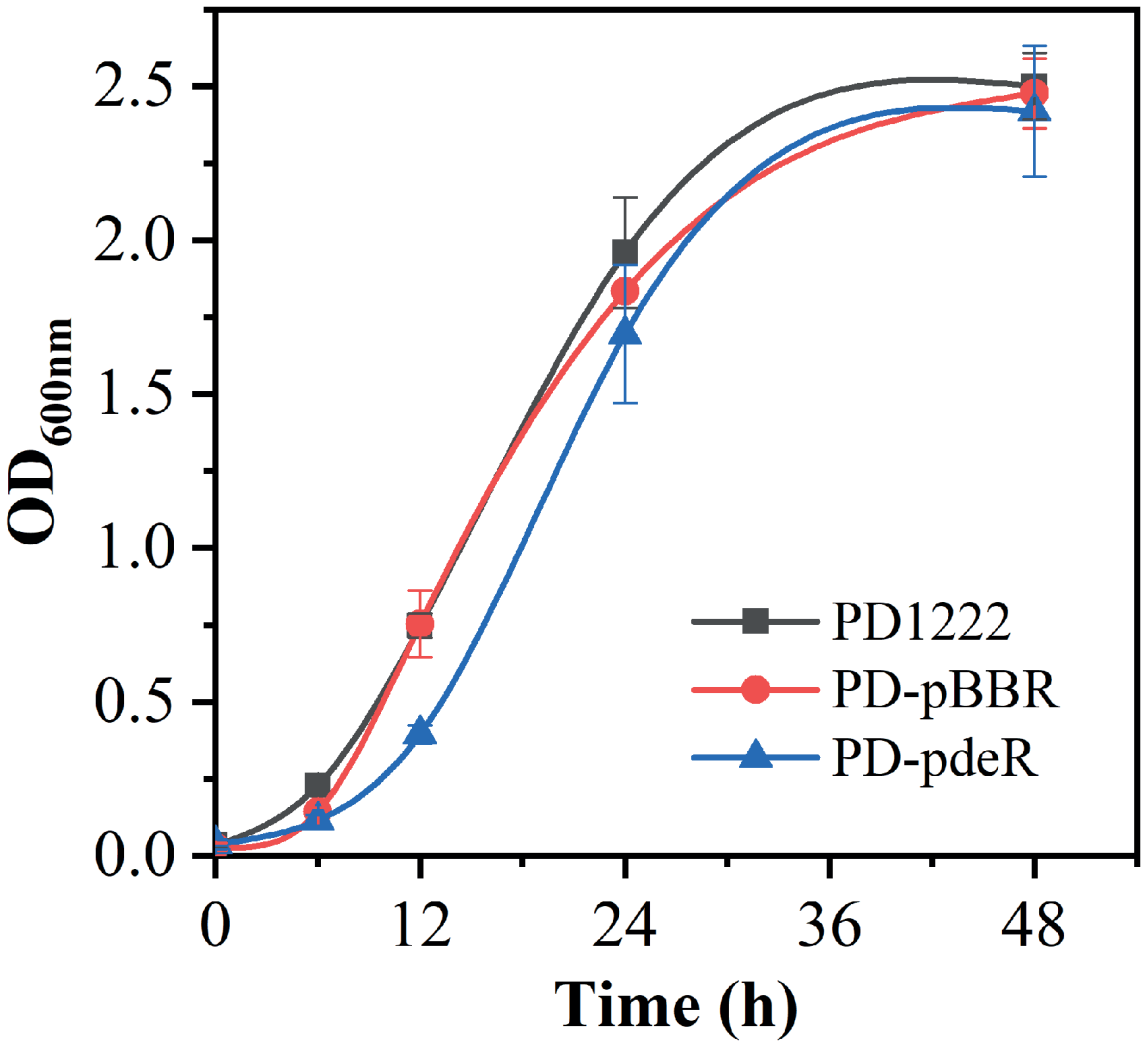
The growth curve of PD1222, PD-pdeR and PD-pBBR. Black, PD122; red, PD-pBBR; blue, PD-pdeR. Data are presented as mean ± SD, *n* = 3.

Recombinant strains PD-pBBR and PD-pdeR were constructed from parent strain *P. denitrificans* PD1222. To validate the overexpression of *pdeR* gene, RT-qPCR was performed to examine the expressional level of pdeR in PD-pBBR, PD-pdeR and PD1222 cells. As shown in Supplementary Figure S1, *pdeR* was up-regulated about 38-fold in PD-pdeR strain comparing with PD1222 cells, while *pdeR* transcription in PD-pBBR strain was comparable to wild type cells. To determine the effect of PdeR protein on *P. denitrificans* growth, PD1222, PD-pBBR and PD-pdeR were seeded into the same medium and incubated for 48 h. The growth curves suggested *pdeR* overexpression slightly inhibited bacterial growth rate and led to a longer lag phase (Figure 1). While the empty plasmid did not show any significant effect on bacterial growth.

**Figure 2:**
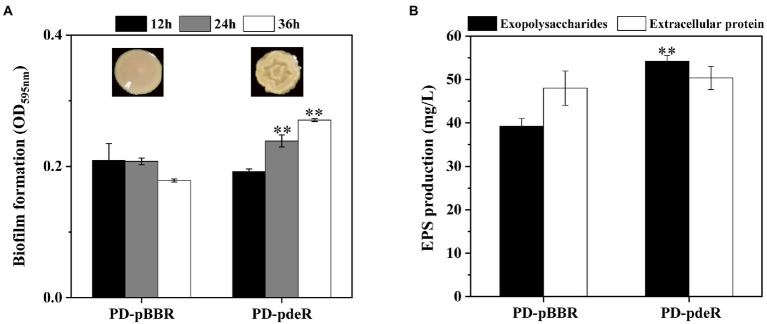
**(A)** The colony morphology and the biofilm formation of PD-pBBR (left) and PD-pdeR (right) strains after 12, 24 and 36 h incubation, respectively. **(B)** The exopolysaccharides and extracellular protein production of PD-pBBR and PD-pdeR after 36 h incubation. Data are presented as mean ± SD, *n* = 3. ^**^p < 0.01 compared with the control strain PD-pBBR.

**Figure 3:**
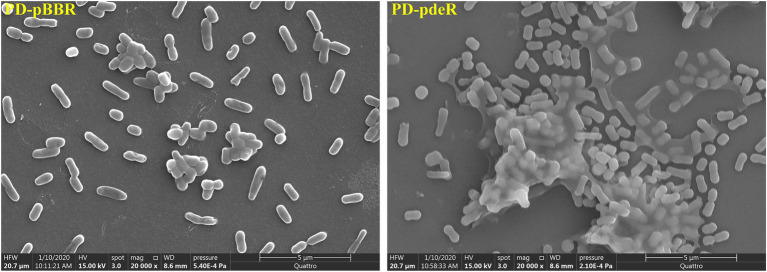
The morphology of PD-pBBR and PD-pdeR using SEM (scale bar = 5 μm). Cells were incubated in LB medium for 36 h, the glass slides that were pre-placed in 24-well plates were directly processed for Scanning Electron Microscope (SEM) observation.

However, the *pdeR* overexpressed strains produced a distinctive colony morphology on the LB agar plate (Figure 2A). The colony of PD-pdeR showed a wrinkled appearance, while the colony surface of PD-pBBR was smoother. In bacteria, wrinkled or rugose colonies are assumed to be a consequence of the formation of biofilms on the colony surface (Mandel and Dunn, 2016), thus we further detected the biofilm formation and EPS production of PD-pBBR and PD-pdeR, respectively. As the results showed, bacteria started to form detectable biofilm after 12 h cultivation, in which enhanced biofilm formation of PD-pdeR strains was observed, especially in 24 and 36 h (Figure 2A). Accordingly, PD-pdeR strains produced more EPS especially exopolysaccharides than PD-pBBR strains after 36 h incubation (Figure 2B). Moreover, as observed by SEM, cells of PD-pdeR had conspicuous aggregation and were covered by extracellular matrix (Figure 3). The results above clearly indicated the role of PdeR protein in the biofilm formation of *P. denitrificans*; however, the molecular mechanisms of PdeR protein involved in biofilm formation remain to be further investigated.

### *pdeR* overexpression altered gene transcription profiles in *Paracoccus denitrificans* PD1222

**Figure 4:**
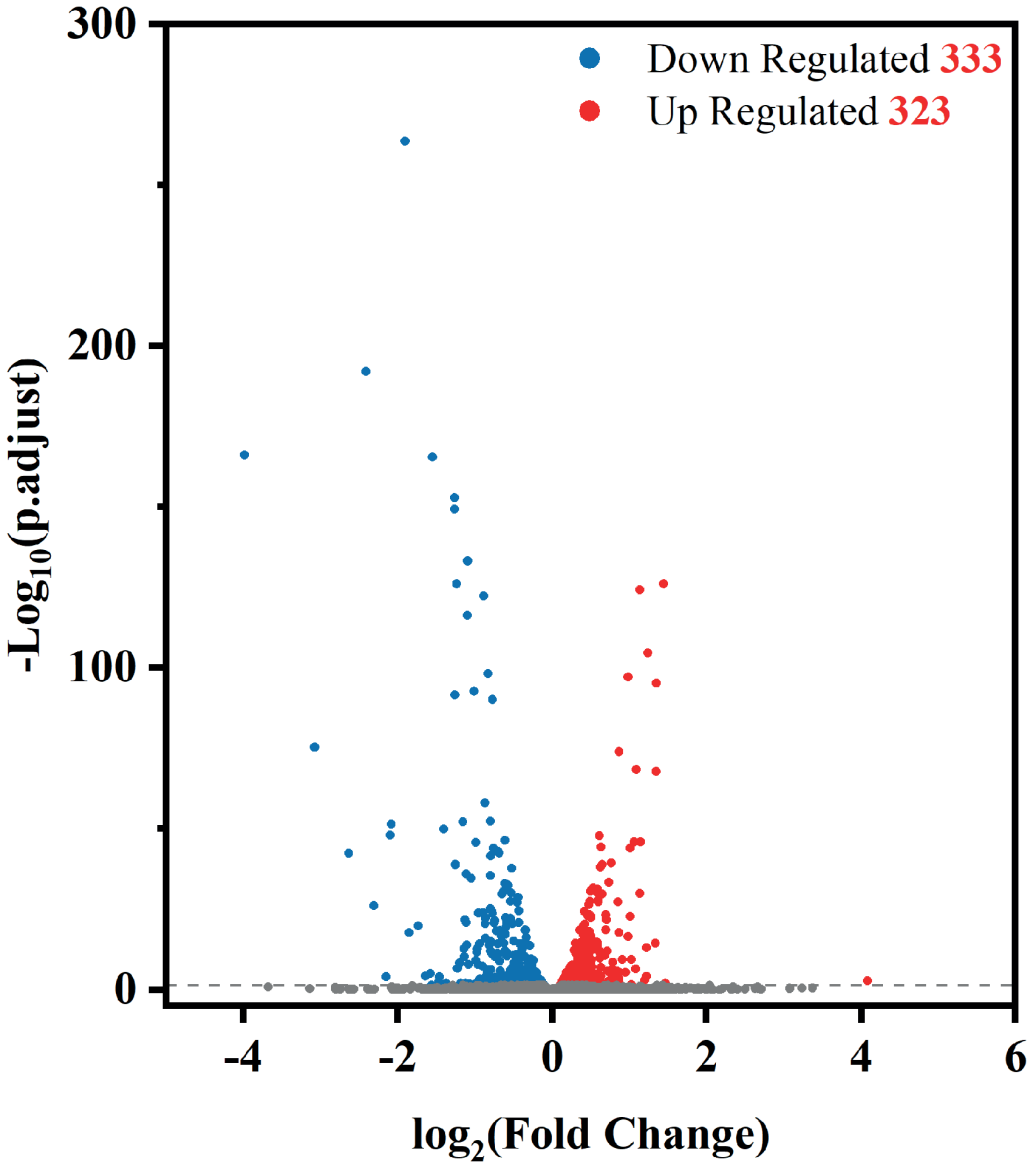
Volcano plot of differentially expressed genes between PD-pdeR and PD-pBBR (red, up-regulated genes; blue, downregulated genes; gray, non-differentially expressed genes).

**Figure 5:**
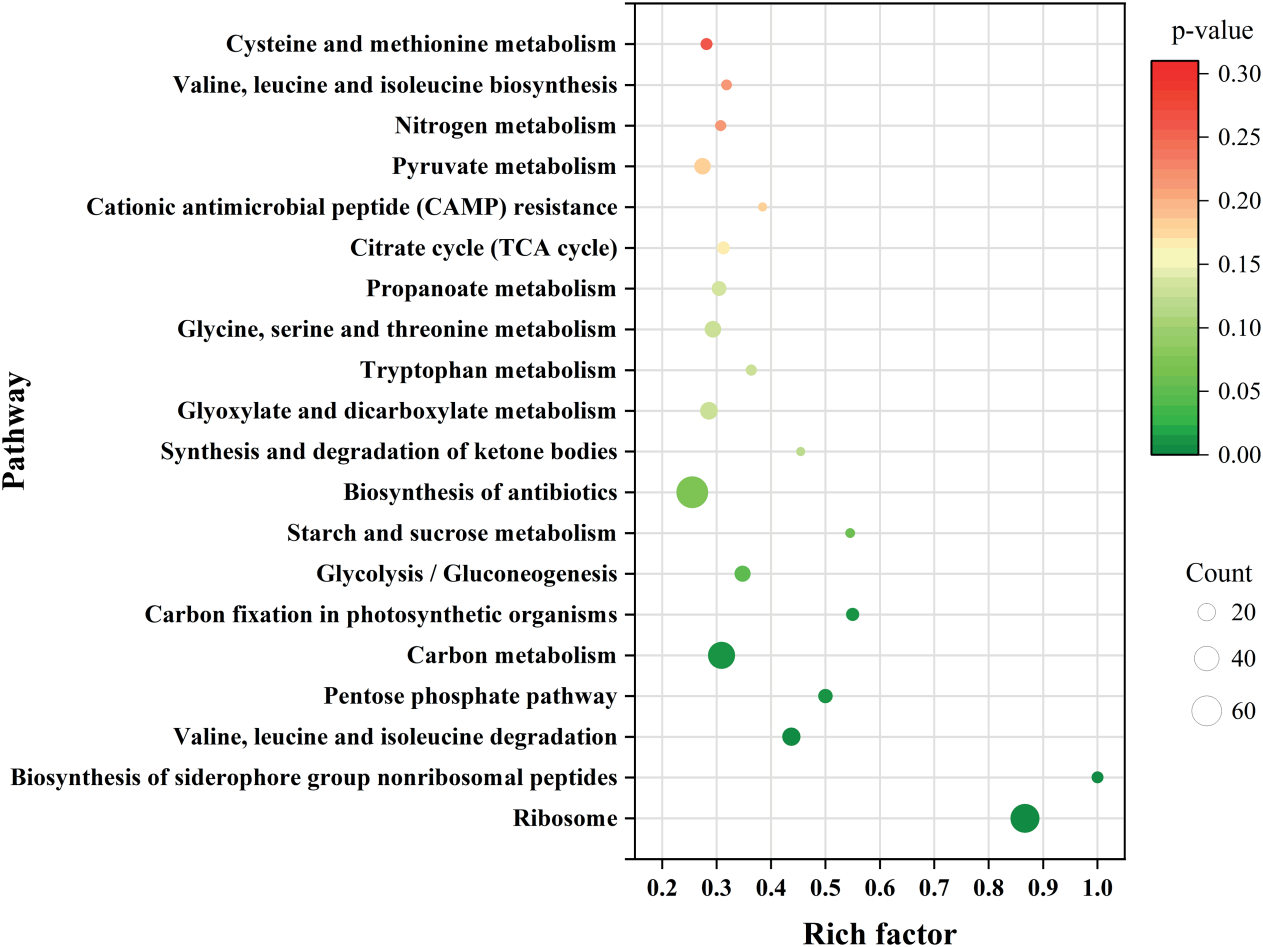
Scatterplot of top 20 KEGG pathway enrichment for differentially expressed genes (DEGs) in PD-pdeR. Data was displayed with the parameters of gene count, gene ratio and value of *p*.

To determine which genes are regulated by PdeR, we performed RNA-seq analysis to collect the DEGs after *pdeR* overexpressed in *P. denitrificans*. As shown in Figure 4, around 656 genes were under the regulation of PdeR, of which 333 genes were downregulated and 323 genes were up-regulated. We further analyzed the enrichment of the KEGG pathways through the KEGG database.^1^ The top 20 KEGG pathways are presented, in which DEGs involved in ribosome and biosynthesis of siderophore group nonribosomal peptides pathway were significantly enriched (Figure 5). Besides, various carbon metabolism pathways such as glycolysis, pyruvate metabolism, tricarboxylic acid cycle (TCA cycle), pentose phosphate pathway, starch and sucrose metabolism were also included. Amino acid metabolism including valine, leucine, isoleucine degradation and tryptophan, glycine, serine, threonine, cysteine and methionine metabolism were enriched. Moreover, the enriched KEGG pathways also included fatty acid metabolism and antibiotics resistance-related pathways (Figure 5). In addition, three genes participating in O-Antigen nucleotide sugar biosynthesis pathway were also significantly up-regulated in PD-pdeR (Supplementary Table S1). Previous studies have demonstrated that nucleotide sugars are the precursors for exopolysaccharide biosynthesis (Padmanabhan et al., 2018). These results suggested that PdeR regulates multiple genes and metabolic pathways, functioning as a global regulator in *P. denitrificans*.

### *pdeR* overexpression promoted ATP synthesis in *Paracoccus denitrificans* PD1222

**Figure 6:**
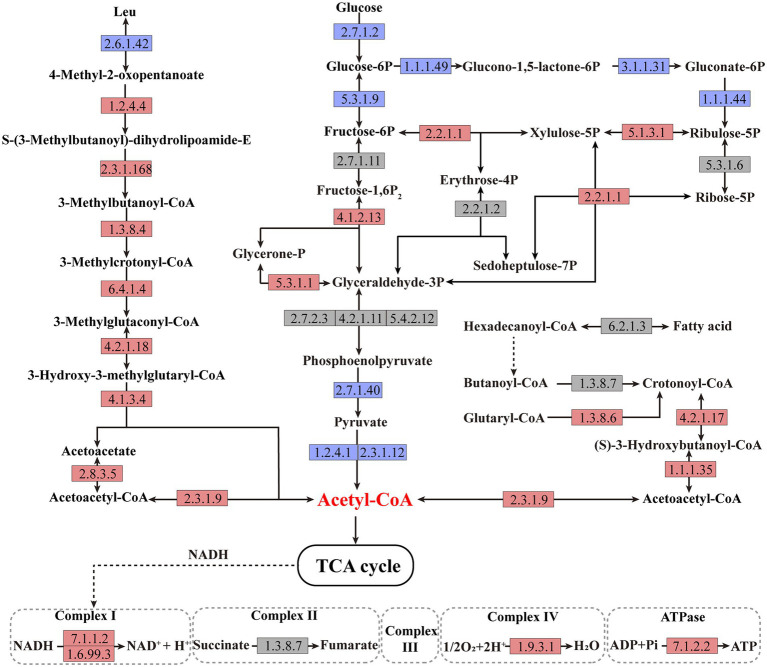
Schematic metabolic pathways of amino acid degradation, fatty acid degradation, central carbon metabolic and oxidative phosphorylation in *Paracoccus denitrificans* per the KEGG pathway database (https://www.genome.jp/kegg/pathway.html). The up-regulated, downregulated, and non-differentially expressed genes of the catalytic enzymes are marked with red, blue, and gray colors, respectively. The detailed different expression values could be found in Supplementary Table S1.

In order to explore the underlying mechanism of PdeR promoting biofilm formation, we performed further in-depth data mining on transcription analysis results, mainly focused on the energy production related pathways, including amino acid degradation, carbon metabolic pathways, fatty acid degradation and oxidative phosphorylation. As shown in Figure 6, taking leucine degradation as an example, leucine is catabolized to acetyl-CoA catalyzing by a series of enzymes, and genes encoding these enzymes were all up-regulated in PD-pdeR (detailed different expression values are listed in Supplementary Table S1), suggesting PdeR promoted amino acids degradation. Meanwhile, fatty acid degradation was promoted as well. The transcriptome analysis indicated the expressional level of key enzymes involved in β-oxidation was up-regulated, including 3-hydroxyacyl-CoA dehydrogenase (EC: 4.2.1.17) and 3-hydroxyacyl-CoA dehydrogenase (EC: 1.1.1.35). The up-regulated amino acids and fatty acids degradation yielded more NADH, FADH_2_ and acetyl-CoA which could enter the TCA cycle and oxidative phosphorylation to produce ATP.

Besides, the central carbon metabolism was also affected by PdeR. First, part of the key enzymes that participated in glycolysis was downregulated, such as glucokinase (EC: 2.7.1.2), pyruvate kinase (EC: 2.7.1.40), and pyruvate dehydrogenase (EC: 1.2.4.1 and EC: 2.3.1.12), and thus, the production of acetyl-CoA deriving from glycolysis was decreased (Figure 6). Besides, enzymes catalyzing critical steps of the pentose phosphate pathway that related to NADPH generation including EC: 1.1.1.49, EC: 3.1.1.31and EC: 1.1.1.44, were all inhibited in PD-pdeR (Figure 6). Nevertheless, the expressional level of genes related to three-, four-, five-, six-and seven-carbon compounds transformation was promoted, such as EC: 2.2.1.1 and EC: 5.1.3.1.

Moreover, enzymes involved in the oxidative phosphorylation pathway were also up-regulated in PD-pdeR, including the NAD-dependent dehydratase (also known as complex I, EC: 7.1.1.2 and EC: 1.6.99.3), the cytochrome c oxidase (also known as complex IV, EC: 1.9.3.1) and ATP synthase (also known as complex V, EC: 7.1.2.2), suggesting the ATP production in PD-pdeR was accelerated (Figure 6).

**Figure 7:**
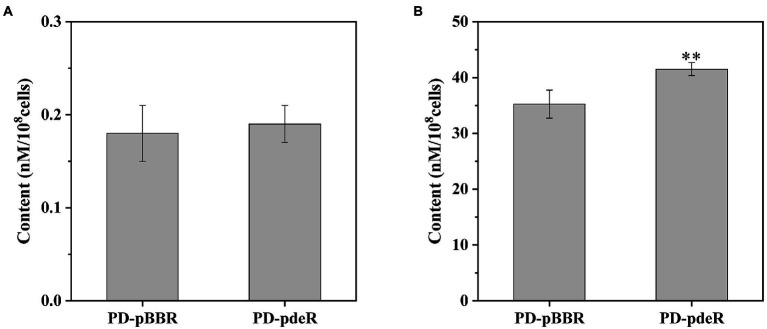
The content of acetyl-CoA **(A)** and ATP **(B)** in PD-pBBR and PD-pdeR. Cells were cultivated in LB medium and collected when OD_600_ was about 1.5. Data are presented as mean ± SD, n = 3. ^**^p < 0.01 compared with the control strain PD-pBBR.

Subsequently, we detected the amount of acetyl-CoA and ATP in PD-pdeR as well as PD-pBBR, respectively. As shown in Figure 7A, neither PD-pdeR nor PD-pBBR had detected significant acetyl-CoA accumulation (all values were less than 1 nM/10^8^ cells). We speculate that this may be because acetyl-CoA would be rapidly consumed after generating (Kremer et al., 2019). In contrast, compared with PD-pBBR, the cells of PD-pdeR contained more ATP (about 41.5 nM/10^8^ cells), suggesting the overexpressed PdeR protein promoted ATP production (Figure 7B).

### *pdeR* overexpression promoted iron absorption in *Paracoccus denitrificans* PD1222

**Figure 8:**
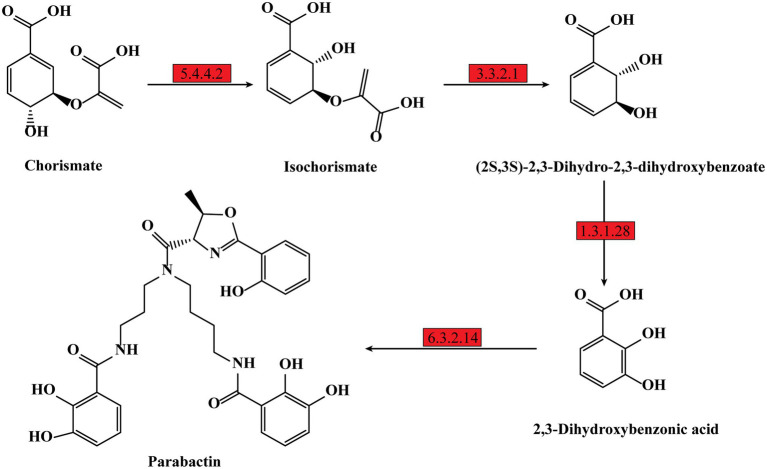
Schematic metabolic pathway of parabactin biosynthesis in *P. denitrificans* per the KEGG pathway database (https://www.genome.jp/kegg/pathway.html). The up-regulated expressed genes of the enzymes are marked with red, and the detailed different expression values could be found in Supplementary Table S2.

**Figure 9:**
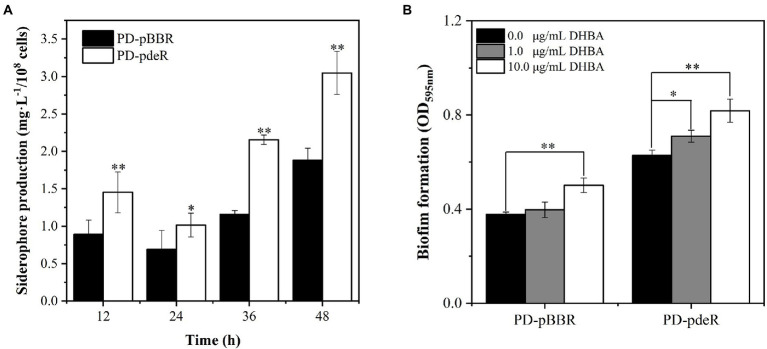
**(A)** Detection of siderophore production of PD-pBBR (black bar) and PD-pdeR (white bar) using Arnow test after 12, 24, 36 and 48 h incubation, respectively. **(B)** The effect of DHBA addition on biofilm formation. The addition concentrations of DHBA were 0, 1 and 10 μg/ml, and biofilm was detected after 36 h incubation. Data are presented as mean ± SD, *n* = 3. ^*^p < 0.05, ^**^p < 0.01 compared with the negative control group.

Previous studies showed iron is essential for bacteria growth as well as biofilm formation (Banin et al., 2005; Kang and Kirienko, 2018). To obtain necessary iron, most bacteria secrete various iron-chelators, called siderophores, to efficiently chelate environmental iron (usually Fe^3+^; Andrews et al., 2003). It has been shown *P. denitrificans* could produce a catechol siderophore, called parabactin, and its derivatives to seize iron from the environment (Tait, 1975). In *P. denitrificans*, parabactin is synthesized from chorismate catalyzed by a series of enzymes, including isochorismate synthase (EC: 5.4.4.2), isochorismatase (EC: 3.3.2.1), 2,3-dihydro-2,3-dihydroxybenzoate dehydrogenase (EC: 1.3.1.28) and nonribosomal peptide synthetase (EC: 6.3.2.14), which were all up-regulated in PD-pdeR strain as shown in Figure 8 (detailed values are displayed in Supplementary Table S1). Moreover, we also found several genes encoding iron transporters were all up-regulated in PD-pdeR, including the TonB-dependent iron transporters, ABC-type iron transporters, and heme transporters (Supplementary Table S2). Next, we quantified the siderophore production of PD-pBBR and PD-pdeR at different growth stages using the Arnow test. Consistent with transcriptomic analysis, during growth, PD-pdeR did yield more siderophore (Figure 9A). Besides, we also examined the effect of 2,3-dihydroxybenzoic acid (DHBA) on bacterial biofilm formation. As the important product of siderophore biosynthesis pathway as well as a powerful iron-chelating molecule, addition of DHBA significantly promoted biofilm formation (Figure 9B). These results showed that *pdeR* overexpression promoted siderophore production and transportation, thus promoted iron absorption to form thicker biofilm.

## Discussion

*Paracoccus denitrificans* is widely spread in either natural or artificial environments, functioning as a driver of the nitrogen cycle (Wang et al., 2021). To survive better, environmental bacteria usually exist in the form of biofilms. And it is well known that biofilm requires QS system regulation for its initiation and maturation (P. Stoodley et al., 2002). Previous studies showed *P. denitrificans* harbors a LuxI/R-type QS circuit, PdeI/R (Zhang et al., 2018). However, little is known about the role and regulation mechanism of QS on biofilm formation of *P. denitrificans* PD1222. In this study, we constructed the *pdeR* overexpression strain and performed phenotypic as well as global transcriptomic studies to provide information on the function of PdeR in *P. denitrificans* PD1222.

Results showed the *pdeR* overexpression strain PD-pdeR produced more EPS and formed thicker biofilm compared with the control strain PD-pBBR (Figures 2, 3). These results are in keeping with previous observational studies, for examples, the *lasI* mutant cells of *P. aeruginosa* could only form flat and undifferentiated biofilms (Davies et al., 1998); and in *Vibrio parahaemolyticus*, the lack of QS regulator protein AphA reduced the EPS production and thus hampered cells’ ability of biofilm formation (Wang et al., 2013). To explore the molecular mechanism of PdeR promoting biofilm formation, we performed transcriptome sequencing analysis. As the results showed, PdeR is a global regulation factor, it could directly or indirectly control the expression of multiple genes which are involved in various metabolic pathways (Figures 4, 5). However, exactly which genes are the key contributing to biofilm initiation needs to be further explored.

In *P. denitrificans*, several molecules have been demonstrated with the ability to affect biofilm formation: that is, the adhesion protein BapA that initiates biofilm formation, the intracellular second messenger cyclic diguanosine monophosphate (Cyclic-di-GMP) and nitric oxide (Kumar and Spiro, 2017; Yoshida et al., 2017). However, these related genes’ expressional levels were not affected by the overexpressed PdeR. Moreover, although three genes participating in O-Antigen nucleotide sugar biosynthesis pathway were up-regulated in PD-pdeR, the expressional level of other genes related to polysaccharide synthesis and flagella or pili assembly was not affected by PdeR much. Remarkably, we found that PdeR promoted a series of metabolic pathways participating in ATP production, including amino acids degradation pathway, fatty acid degradation pathway and oxidative phosphorylation pathway (Figure 6). Similarly to our findings, Pisithkul et al. had also reported during biofilm development in *Bacillus subtilis*, several metabolic alterations which were hitherto unrecognized as biofilm-associated had been detected, such as the tricarboxylic acid (TCA) cycle, fatty acid biosynthesis and degradation, etc. (Pisithkul et al., 2019). These results suggested bacteria tend to promote the degradation of organic substances and accelerate the production of energy during biofilm formation. This is understandable given the fact that biofilm formation needs a large number of extracellular polysaccharides, proteins and a wide variety of secondary metabolites (Payne and Boles, 2016). The biosynthesis and transportation of these molecules are both energy-consuming, thus cells need to generate enough energy to support the synthesis and secretion of these essential substances (Sutherland, 2001). Consistently, we indeed detected higher ATP concentration in PD-pdeR strain cells, indicating PdeR promoted ATP production and thus benefit bacterial biofilm formation.

In addition to energy, siderophore, as the main tool bacteria used to obtain iron is also essential for bacterial growth as well as biofilm formation. Previous studies showed the growth rate of *B. subtilis* mutant strains that lack the function of siderophore biosynthesis was significantly inhibited (Rizzi et al., 2019). Qin et al. demonstrated that the siderophore biosynthetic genes mutant cells of *B. subtilis* showed reduced biofilm formation, while the addition of exogenous iron chelator DHBA could restore mutant strains’ biofilm formation (Qin et al., 2019). And in *B. cepacia*, the production of siderophore ornibactin was affected by the *luxR* homolog *cepR* (Lewenza et al., 1999). Our results in this work are in accord with these previous studies indicating that the increased siderophore production and iron transportation is another key point of PdeR promoting bacterial biofilm formation.

In conclusion, PdeR plays a key role in promoting *P. denitrificans* biofilm formation, mainly through accelerating ATP production and increasing iron transportation at the initiation period. These data brought information about detailed mechanisms that may at least in part explain how QS regulates biofilm formation of *P. denitrificans*, adding to our understanding of QS regulation in biofilm development.

## Data availability statement

The datasets presented in this study can be found in online repositories. The names of the repository/repositories and accession number(s) can be found at: https://www.ncbi.nlm.nih.gov/, bioproject: PRJNA847411.

## Author contributions

NW and JG conceived and designed the experiments. NW and SX performed the experiments and carried out the analysis. NW drafted the manuscript. JG and GZ revised the manuscript. All authors were involved in the discussion of the results and in writing the manuscript. All authors contributed to the article and approved the submitted version.

## Funding

This work was supported by the Beijing Natural Science Foundation (no. 5222025), the National Key Research and Development Program of China (no. 2018YFA090024-04) and the National Natural Science Foundation of China (no. 41501250).

## Conflict of interest

The authors declare that the research was conducted in the absence of any commercial or financial relationships that could be construed as a potential conflict of interest.

## Publisher’s note

All claims expressed in this article are solely those of the authors and do not necessarily represent those of their affiliated organizations, or those of the publisher, the editors and the reviewers. Any product that may be evaluated in this article, or claim that may be made by its manufacturer, is not guaranteed or endorsed by the publisher.

## Footnotes

^1^ https://www.genome.jp/kegg
