# Supplementary material for: Overexpression of pdeR promotes biofilm formation of Paracoccus denitrificans by promoting ATP production and iron acquisition
Source: Front Microbiol. 2022 Aug 10;13:966976. doi: 10.3389/fmicb.2022.966976 (PMC9399729; doi:10.3389/fmicb.2022.966976)
Supplement: Supplementary file 1 [file Table_1.DOCX]

**Table S1** The differently expressed genes involved in multiple metabolic pathways.

| **Gene ID** | **PD-pdeR/PD-pBBR Fold Change** | **p.adjust** | **Annotation** | EC number |
| --- | --- | --- | --- | --- |
| Pden_2602 | 0.769 | 0.0000 | branched-chain amino acid amino transferase | 2.6.1.42 |
| Pden_4757 | 1.367 | 0.0000 | 3-methyl-2-oxobutanoate dehydrogenase(2-methylpropanoyl-transferring) subunit alpha | 1.2.4.4 |
| Pden_4758 | 1.446 | 0.0000 | alpha-keto acid dehydrogenase subunit beta |  |
| Pden_4759 | 1.529 | 0.0000 | 2-oxo-acid dehydrogenase subunit E2 | 2.3.1.168 |
| Pden_3633 | 1.201 | 0.0000 | isovaleryl-CoA dehydrogenase | 1.3.8.4 |
| Pden_3637 | 1.176 | 0.0002 | methylcrotonoyl-CoA carboxylase | 6.4.1.4 |
| Pden_3639 | 1.119 | 0.0271 | ATP-grasp domain-containing protein |  |
| Pden_3642 | 1.296 | 0.0014 | enoyl-CoA hydratase | 4.2.1.18 |
| Pden_2871 | 1.195 | 0.0000 | 3-hydroxyacyl-CoA dehydrogenase | 4.1.3.4 |
| Pden_4683 | 1.427 | 0.0000 | CoA transferase subunit A | 2.8.3.5 |
| Pden_4684 | 1.401 | 0.0000 | CoA transferase subunit B |  |
| Pden_2907 | 1.507 | 0.0000 | acetyl-CoA C-acyl transferase | 2.3.1.9 |
| Pden_2870 | 1.208 | 0.0012 | acetyl-CoA acetyltransferase |  |
| **Val, Leu, Ile degradation** | | | | |
| Pden_2680 | 0.623 | 0.0000 | glucokinase | 2.7.1.2 |
| Pden_1950 | 0.617 | 0.0000 | glucose-6-phosphate isomerase | 5.3.1.9 |
| Pden_1698 | 1.736 | 0.0000 | fructose-bisphosphate aldolase class II | 4.1.2.13 |
| Pden_1920 | 1.359 | 0.0000 | fructose-bisphosphate aldolase |  |
| Pden_4305 | 1.516 | 0.0000 | triose-phosphate isomerase | 5.3.1.1 |
| Pden_2276 | 0.720 | 0.0000 | pyruvatekinase | 2.7.1.40 |
| Pden_3890 | 0.791 | 0.0000 | pyruvate dehydrogenase complex dihydrolipoamide acetyl transferase | 1.2.4.1 |
| Pden_3891 | 0.763 | 0.0000 | pyruvate dehydrogenase complex E1 component subunit beta |  |
| Pden_3892 | 0.805 | 0.0000 | pyruvate dehydrogenase(acetyl-transferring) E1 component subunit alpha | 2.3.1.12 |
| **Glycolysis** | | | | |
| Pden_1951 | 0.649 | 0.0049 | 6-phosphogluconolactonase | 1.1.1.49 |
| Pden_1952 | 0.611 | 0.0000 | glucose-6-phosphate dehydrogenase | 3.1.1.31 |
| Pden_2393 | 0.611 | 0.0000 | phosphogluconate dehydrogenase (NADP (+)-dependent decarboxylating) | 1.1.1.44 |
| Pden_2572 | 1.115 | 0.0293 | ribulose-phosphate3-epimerase | 5.1.3.1 |
| Pden_1697 | 1.639 | 0.0001 | transketolase | 2.2.1.1 |
| Pden_2499 | 0.852 | 0.0000 | transaldolase | 2.2.1.2 |
| **Pentose phosphate pathway** | | | | |
| Pden_2871 | 1.195 | 0.0000 | 3-hydroxyacyl-CoA dehydrogenase | 1.1.1.35 |
|  |  |  |  | 4.2.1.17 |
| Pden_2907 | 1.507 | 0.0000 | acetyl-CoA C-acyltransferase | 2.3.1.9 |
| Pden_2870 | 1.208 | 0.0012 | acetyl-CoA acetyltransferase |  |
| Pden_4412 | 1.743 | 0.0000 | acyl-CoA dehydrogenase | 1.3.8.6 |
| **Fatty acid degradation** | | | | |
| Pden_0492 | 1.119 | 0.0024 | complex IND UFA9 subunit family protein | 7.1.1.2 |
| Pden_1845 | 1.272 | 0.0000 | cbb3-type subunit III | 1.9.3.1 |
| Pden_1847 | 1.240 | 0.0000 | cbb3-type subunit II |  |
| Pden_1848 | 1.243 | 0.0000 | cbb3-type subunit I |  |
| Pden_3815 | 1.084 | 0.0365 | ATP synthase subunit delta | 7.1.2.2 |
| Pden_3816 | 1.099 | 0.0018 | ATP synthase subunit alpha |  |
| Pden_3817 | 1.090 | 0.0072 | ATP synthase subunit gamma |  |
| Pden_3818 | 1.096 | 0.0028 | ATP synthase subunit beta |  |
| Pden_3819 | 1.107 | 0.0035 | ATP synthase epsilon chain |  |
| **Oxidative phosphorylation** | | | | |
| Pden_2382 | 1.983 | 0.0000 | isochorismate synthase | 5.4.4.2 |
| Pden_2383 | 1.686 | 0.0000 | isochorismatase family protein | 3.3.2.1 |
| Pden_2384 | 1.528 | 0.0000 | 2,3-dihydro-2,3-dihydroxybenzoate dehydrogenase | 1.3.1.28 |
| Pden_2386 | 1.573 | 0.0000 | (2,3-dihydroxybenzoyl) adenylate synthase | 6.3.2.14 |
| Pden_2387 | 2.204 | 0.0000 | condensation protein |  |
| **Biosynthesis of siderophore group nonribosomal peptides** | | | | |
