# Supplementary material for: Overexpression of pdeR promotes biofilm formation of Paracoccus denitrificans by promoting ATP production and iron acquisition
Source: Front Microbiol. 2022 Aug 10;13:966976. doi: 10.3389/fmicb.2022.966976 (PMC9399729; doi:10.3389/fmicb.2022.966976)
Supplement: Supplementary file 2 [file Table_2.DOCX]

**Table S2** DEGs of iron transport system.

| **Gene ID** | **PD-pdeR/PD-pBBR FC** | **Annotation** |
| --- | --- | --- |
| Pden_0296 | 2.121 | TonB-dependent receptor |
| Pden_0299 | 1.776 | iron ABC transporter permease |
| Pden_0300 | 1.760 | iron ABC transporter substrate-binding protein |
| Pden_0725 | 1.714 | TonB-system energy transducer |
| Pden_0726 | 1.734 | TonB system transport protein ExbD |
| Pden_0727 | 1.741 | TonB-system energizer ExbB |
| Pden_1139 | 1.612 | TonB-dependent siderophore receptor |
| Pden_1733 | 1.671 | iron transporter |
| Pden_1734 | 1.630 | iron transporter substrate-binding protein |
| Pden_1735 | 1.686 | deferrochelatase EfeB |
| Pden_1736 | 1.708 | EfeM/EfeO family lipoprotein |
| Pden_1775 | 1.838 | TonB-dependent siderophore receptor |
| Pden_2046 | 2.933 | TonB-dependent receptor |
| Pden_3007 | 2.404 | TonB-dependent receptor |
| Pden_3008 | 1.959 | 2,2 C3-dihydroxybenzoate-AMP ligase |
| Pden_3009 | 2.167 | chorismate mutase related enzymes |
| Pden_3010 | 1.671 | ABC transporter substrate-binding protein |
| Pden_3011 | 1.980 | iron ABC transporter permease |
| Pden_3521 | 1.762 | TonB-dependent siderophore receptor |
| Pden_3522 | 1.234 | iron-siderophore ABC transporter substrate-binding protein |
| Pden_4201 | 2.642 | TonB-dependent receptor |
| Pden_4202 | 2.160 | hemin-degrading factor |
| Pden_4203 | 1.967 | hemin ABC transporter substrate-binding protein |
| Pden_4204 | 2.151 | iron ABC transporter permease |
| Pden_4205 | 2.285 | heme ABC transporter ATP-binding protein |
