# Supplementary material for: Overexpression of pdeR promotes biofilm formation of Paracoccus denitrificans by promoting ATP production and iron acquisition
Source: Front Microbiol. 2022 Aug 10;13:966976. doi: 10.3389/fmicb.2022.966976 (PMC9399729; doi:10.3389/fmicb.2022.966976)
Supplement: Supplementary file 3 [file Figure_S1.DOCX]

Figure S1. The expression difference of *pdeR* inPD1222, PD-pBBR and PD-pdeR strains. Data are presented as mean ± SD. n = 3. ***P* < 0.01 compared with the wild type strain PD1222.
